# An ontology approach to comparative phenomics in plants

**DOI:** 10.1186/s13007-015-0053-y

**Published:** 2015-02-25

**Authors:** Anika Oellrich, Ramona L Walls, Ethalinda KS Cannon, Steven B Cannon, Laurel Cooper, Jack Gardiner, Georgios V Gkoutos, Lisa Harper, Mingze He, Robert Hoehndorf, Pankaj Jaiswal, Scott R Kalberer, John P Lloyd, David Meinke, Naama Menda, Laura Moore, Rex T Nelson, Anuradha Pujar, Carolyn J Lawrence, Eva Huala

**Affiliations:** Wellcome Trust Sanger Institute, Wellcome Trust Genome Campus, Hinxton, CB10 1SA UK; iPlant Collaborative, University of Arizona, 1657 E. Helen St., Tucson, Arizona 85721 USA; Department of Electrical and Computer Engineering Iowa State University, 1018 Crop Informatics Lab, Ames, Iowa 50011 USA; USDA-ARS Corn Insects and Crop Genetics Research Unit, Iowa State University, Crop Genome Informatics Lab, Iowa State University, Ames, IA 50011 USA; Department of Agronomy, Agronomy Hall, Iowa State University, Ames, IA 50010 USA; Department of Botany and Plant Pathology, 2082 Cordley Hall, Oregon State University, Corvallis, OR 97331 USA; Department of Genetics, Development and Cell Biology, Roy J Carver Co-Laboratory, Iowa State University, Ames, IA 50010 USA; Department of Computer Science, Aberystwyth University, Llandinam Building, Aberystwyth, SY23 3DB UK; Computer, Electrical and Mathematical Sciences & Engineering Division and Computational Bioscience Research Center, King Abdullah University of Science and Technology, 4700 King Abdullah University of Science and Technology, P.O. Box 2882, Thuwal, 23955-6900 Kingdom of Saudi Arabia; Department of Plant Biology, Michigan State University, 220 Trowbridge Rd, East Lansing, MI 48824 USA; Department of Botany, Oklahoma State University, 301 Physical Sciences, Stillwater, OK 74078 USA; Boyce Thompson Institute for Plant Research, 533 Tower Road, Ithaca, NY 14853 USA; Phoenix Bioinformatics, 643 Bair Island Rd Suite 403, Redwood City, CA 94063 USA

## Abstract

**Background:**

Plant phenotype datasets include many different types of data, formats, and terms from specialized vocabularies. Because these datasets were designed for different audiences, they frequently contain language and details tailored to investigators with different research objectives and backgrounds. Although phenotype comparisons across datasets have long been possible on a small scale, comprehensive queries and analyses that span a broad set of reference species, research disciplines, and knowledge domains continue to be severely limited by the absence of a common semantic framework.

**Results:**

We developed a workflow to curate and standardize existing phenotype datasets for six plant species, encompassing both model species and crop plants with established genetic resources. Our effort focused on mutant phenotypes associated with genes of known sequence in *Arabidopsis thaliana* (L.) Heynh. (Arabidopsis), *Zea mays* L. subsp. *mays* (maize), *Medicago truncatula* Gaertn. (barrel medic or Medicago), *Oryza sativa* L. (rice), *Glycine max* (L.) Merr. (soybean), and *Solanum lycopersicum* L. (tomato). We applied the same ontologies, annotation standards, formats, and best practices across all six species, thereby ensuring that the shared dataset could be used for cross-species querying and semantic similarity analyses. Curated phenotypes were first converted into a common format using taxonomically broad ontologies such as the Plant Ontology, Gene Ontology, and Phenotype and Trait Ontology. We then compared ontology-based phenotypic descriptions with an existing classification system for plant phenotypes and evaluated our semantic similarity dataset for its ability to enhance predictions of gene families, protein functions, and shared metabolic pathways that underlie informative plant phenotypes.

**Conclusions:**

The use of ontologies, annotation standards, shared formats, and best practices for cross-taxon phenotype data analyses represents a novel approach to plant phenomics that enhances the utility of model genetic organisms and can be readily applied to species with fewer genetic resources and less well-characterized genomes. In addition, these tools should enhance future efforts to explore the relationships among phenotypic similarity, gene function, and sequence similarity in plants, and to make genotype-to-phenotype predictions relevant to plant biology, crop improvement, and potentially even human health.

**Electronic supplementary material:**

The online version of this article (doi:10.1186/s13007-015-0053-y) contains supplementary material, which is available to authorized users.

## Background

Plant phenotypic variation constitutes the raw material for much of plant biology, including research on gene function in model species, breeding of desirable crop varieties, functional investigations from the cellular to ecosystem scale, and inference about the evolution and ecology of both plants and the species that interact with them. Disentangling the relationships among genotypes, phenotypes, and the environment is one of the grand challenges of contemporary biology [1], yet this endeavor is severely limited by our ability to collect, integrate, and systematically analyze phenotypic data [2]. Researchers generally use free text to describe phenotypes, which allows for rich descriptions, but makes it hard to compare phenotypes across species, integrate data into the existing knowledge landscape, or derive information from combined datasets [3]. In recent years, ontologies have become powerful tools for working with phenotypic data, particularly in biomedicine, because standardizing terminology across species and sub-disciplines enables inference based on logical relationships [4-6]. Here we present a new approach to studying plant phenotypes modeled on recent advances in the use of ontologies in biomedical research on animal model systems.

Throughout this paper, we use the words phenotype, phene, and phenome with precise meanings. A ‘phenotype’ is the composite set of one or more observable characteristics associated with a given organism or cell, that results from the interaction of the genotype and the environment [7,8]. The separate characteristics that make up a phenotype are termed ‘phenes’ [9,10]. For example, in maize, a *dwarf* phenotype can be defined as a composite of the phenes ‘reduced internode length’ and ‘compact, broad leaves’. Phenes relate to ‘phenomes’ in the way that genes relate to genomes: an organism’s or species’ phenome is composed of the complete set of its phenes. Phenomics, therefore, is the study of all phenotypes associated with an organism or species (i.e. its phenotype space). In correspondence with Genome Wide Association Studies (GWAS), Phenome Wide Association Studies (PheWAS) associate a gene with a variety of phenes or phenotypes, which is particularly relevant for genes that have a pleiotropic effect [11].

Biomedical scientists have developed and utilized phenotype ontologies and ontological reasoning to support comparative and predictive phenomics [12,13]. Phenotype ontologies are controlled, hierarchically-related phenotypic descriptions that enable large-scale computation among individuals, populations, and even multiple species [14]. A number of vocabularies and pre-composed phenotype ontologies (in which terms are pre-defined) have been developed for specific taxa or applications [15-18], but comparison across datasets or among different species requires an extensive alignment process whenever different vocabularies/ontologies are used to represent the data. An alternative to phenotype ontology alignment is the use of post-composed phenotypes, in which all the elements of a phenotype are explicitly logically defined or “composed” from existing terms from species-independent ontologies [16]. One method of post-composing a phenotype description is to first break it down into its component phenes, and then define an affected Entity (E) and a describing Quality (Q) for each phene [19,20]. In this method of post-composing phenotypes, Entity-Quality (EQ) statements are composed for all phenes under considerations, and the entire set of phenes is reasoned over simultaneously. Finally, to derive novel insights from curated genotype and phenotype data, semantic similarity measures are applied, based on a consistent ontological representation [21-23].

This approach has been applied successfully to mammalian phenotypes to predict gene function across species, as well as disease, drug, or pathway involvement of genes [5,12,13,24,25]. Two major limitations to adopting a similar approach in plants are the lack of phenotype data curated with species-neutral ontology terms, and the need for standards for creating EQ statements to describe plant phenotypes. Nonetheless, two important existing resources are available to support post-composed ontology analysis of plant phenotype data: 1) well-developed ontologies for plant science [26], particularly the Plant Ontology (PO) [27] and Gene Ontology (GO) [28,29]; 2) curated sets of mutant phenotype descriptions for multiple plant species in model-organism and crop databases such as MaizeGDB [30,31], Oryzabase [32], Gramene [33,34], and the Sol Genomics Network (SGN) [15,35] as well as in the literature (e.g., [36]). In addition, an intellectual framework for logically defining plant traits has been developed in the Plant Trait Ontology (TO) [33].

To push the field of plant phenomics forward, it is clear that there is a need for additional high-quality phenotype descriptions generated by research, as well as for high-confidence predictions of phenotypic associations among equivalent phenotypes, both across species and between phenotypes and their causative genotypic variants and environments. Here we describe how our work to translate existing high-quality phenotypic descriptions across six plant species enabled the prediction of phenotypic associations. Furthermore, we demonstrate that additional curation of such data into ontological representations can expand the phenotypic predictive capacity of plant sciences.

This paper includes methodology, as well as an initial dataset that was used to test and refine the methodology. In brief, we compiled EQ statements for 1,742 phenes from 2,747 genes and gene models in six plant species [*Arabidopsis thaliana* (Arabidopsis), *Zea mays* ssp. *mays* (maize), *Medicago truncatula* (barrel medic or Medicago), *Oryza sativa* (rice), *Glycine max* (soybean), and *Solanum lycopersicum* (tomato)] and applied consistency checks to ensure a high-quality phenotype annotation set. The annotated phenotype data set was subjected to an automated semantic similarity analysis, based on PhenomeNET [37,38], and the results are available in a separate plant instance [39]. The semantic similarity dataset was evaluated for its ability to enhance predictions of gene families, gene functions, and shared metabolic pathways across the six species and compared to an existing classification of plant phenotypes [36].

## Results and discussion

### A method for describing phenotypes with a common semantic representation across six plant species

We include in the Results a brief description of our method, because this is the first report outlining this type of analysis of phenotypes across multiple reference species in plants. For this analysis we limited our species set to the model/crop species Arabidopsis, maize, Medicago, rice, soybean, and tomato, to take advantage of the existing data for these species. However, the method could be applied to any plant for which there are characterized mutant phenotypes associated with sequenced genes. To maximize the ability to compare both phenotypes and genotypes across species, we used only genotypes for which the sequence was known and made efforts to limit our datasets to phenotypes resulting from mutations to a single gene. For genes where phenotype information was available for different alleles, we counted each allele as a separate genotype (Additional file 1). For each species, the authors with the most relevant expertise selected free text phenotype descriptions for inclusion using methods specific to that species (see Methods). The number of genotypes analyzed varied widely among species (maximum 2,393 in Arabidopsis, minimum 30 in soybean), reflecting the availability of phenotypic descriptions for each species.

**Figure 1:**
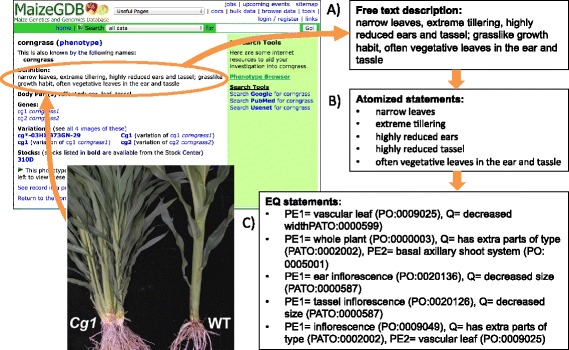
**The method applied to annotate mutant phenotypes from textual descriptions.** Textual descriptions from the literature or databases **(A)**, based on observations of mutant plants, are first broken down into atomized statements corresponding to phenes **(B)** that are then represented with EQ statements **(C)**.

We first decomposed each free text phenotype description into a set of simple atomized statements corresponding to each component, or “phene”, of the phenotype. We then translated each of these components into an EQ (Entity-Quality) statement (Figure 1). As with EQ statements previously developed for mammalian species, we distinguish between structural phenotypes, such as “short plant”, and process phenotypes, such as “late flowering” [40]. In a structural phenotype, the Entity is an affected part of the plant, represented with a term from the Plant Ontology (PO) [27] or Gene Ontology (GO) *cellular component* branch [29]. In a process phenotype, the Entity is an altered process represented with a term from the GO *biological process* branch. In both cases, the manner in which the entity is affected was described using Quality terms from the Phenotype and Trait Ontology (PATO) [16]. For example, the atomized statement “short leaves” can be expressed as: Entity = *vascular leaf* from the Plant Ontology (PO:0009025)^a^ and Quality = *decreased length* from the Phenotype and Trait Ontology (PATO:0000574).

We found that many of the phenes required more complex EQ statements and terms from additional ontologies to fully represent their meaning. For example, the phene “lack of anthocyanins in aleurone” could be expressed as: *aleurone layer* (PO:0005360) *lacks parts or has fewer parts of type* (PATO:0001999) *anthocyanins* (CHEBI:38697), with the form:

$$ \mathrm{primary}\ \mathrm{E}1 - \mathrm{Q} - \mathrm{secondary}\ \mathrm{E}1 $$

where Q is a relational quality. In addition, we found that the Entities themselves may be complex. For example, an EQ statement for the free text “silks are green” would be phrased: *style* (PO:0009074) *part_of* (BFO:0000050)^b^*ear inflorescence* (PO:0020136) *green* (PATO:0000320), with the form:

$$ \left[\mathrm{primary}\ \mathrm{E}1 - \mathrm{R} - \mathrm{primary}\ \mathrm{E}2\right]\ \hbox{--}\ \mathrm{Q} $$

where R is a relation from the Relation Ontology (RO) [41,42]. All EQ statements in the dataset could be parsed using the generalized formula:

$$ \begin{array}{l}\left[\left(\mathrm{primary}\ \mathrm{E}1\right) - \mathrm{R} - \left(\mathrm{primary}\ \mathrm{E}2\right)\right] - \left[\mathrm{Q} - \mathrm{Q}\mathrm{L}\right] - \Big[\left(\mathrm{secondary}\ \mathrm{E}1\right) - \mathrm{R}\ \\ {}-\left(\mathrm{secondary}\ \mathrm{E}2\right)\Big]\end{array} $$

where QL is a qualifier to the quality Q. At a minimum, we required that a primary E1 and Q be present, and any of the other elements were optional. We found that all phenotypes in our dataset could be described with the ontologies listed in Table 1, although we recognize that our dataset does not encompass the entire breadth of possible plant phenotypes, and additional ontologies and development of existing ontologies will be needed to annotate more diverse phenotypes.

**Table 1:** **Description of applied ontologies**

**Ontology**	**Content**	**Link**
Plant Ontology (PO) [27]	Plant anatomy and morphology and development stages	http://www.plantontology.org/
Gene Ontology (GO) [29]	Biological processes, cellular components and molecular functions	http://geneontology.org/
Chemical Entities of Biological Interest ontology (ChEBI) [43]	Molecular entities focused on ‘small’ chemical compounds.	http://www.ebi.ac.uk/chebi/
Phenotypic Qualities Ontology (PATO) [16,19]	Phenotypic qualities	http://obofoundry.org/wiki/index.php/PATO:Main_Page
Plant Experimental Conditions Ontology (EO)	Treatments, growing conditions, and/or study types	http://planteome.org/amigo/cgi-bin/crop_amigo/term_details?term=EO:0007359
NCBI taxonomy (NCBITAXON)	A curated classification and nomenclature for all of the organisms in the public sequence databases.	http://www.ncbi.nlm.nih.gov/taxonomy
Relation Ontology (RO) [41]	Core upper-level relations and biology-specific relations	https://code.google.com/p/obo-relations/

Species-independent ontologies used to form EQ statements. All ontologies were downloaded on 15 March 2014.

Because a phenotype consists of one or more phenes, one or more EQ statements were used to describe it. For example, the phenotype “corngrass” in maize is described as “narrow leaves, extreme tillering, highly reduced ears and tassel; grasslike growth habit, often vegetative leaves in the ear and tassel”. This phenotype was broken down into 5 phenes, which were annotated with EQ statements (Figure 1). Likewise, an individual EQ statement can be used to describe more than one phenotype. In the corngrass example, the EQ statement that describes the “narrow leaf” phene is also used in several other phenotypes, such as “narrowleaf” (Additional file 1). Our approach considers each “phenotype” as the sum of its individual EQ statements.

This method allows for highly detailed and species-independent descriptions of phenotypes, but still has several limitations. Creating accurate EQ statements requires knowledge of both the species-specific phenotypes as well as the ontologies used to describe them. Brief phenotype descriptions may be available in databases (e.g., MaizeGDB, SGN), but much more complete and accurate descriptions of mutant phenotypes are spread over many publications spanning several years. Collecting and extracting phenotype information is very labor intensive. In addition, EQ statements are created by curators and thus still reflect a certain amount of subjectivity. It is often possible to build more than one EQ statement for the same textual description, and it is not always clear when to use a process entity versus a structural entity. In this project, we established a strict set of rules and protocols and held regular meetings to help ensure consistent construction of comparable EQ statements across species (see Methods). In the future, we would like to evaluate the importance of consistently structured EQ statements for analyzing semantic similarity, and determine whether some variation can be tolerated.

### An ontology-based dataset of mutant phenotypes for six reference plant species

The complete list of genes, genotypes, phenotypes, atomized statements, and EQ statements can be found in Additional file 1, which is also included as part of the complete dataset housed in the iPlant Data Commons [44]. The largest set of annotations came from Arabidopsis, followed by maize, rice, and tomato (Table 2). The low numbers of annotations for Medicago and soybean reflect the relatively small number of studies on these species and the scarcity of curated phenotypes. There was little overlap of unique phenotypes among species (Additional file 2). In the following sections, we describe some of types of computational analyses that can be done with this dataset.

**Table 2:** **The number of EQ statements, genes, genotypes, and phenotypes they were associated with, for six plant species**

**Species**	**#EQs (phenes)**	**#unique EQs - all genotypes**	**#genes**	**#genotypes**	**#phenotypes**
*Arabidopsis thaliana*	5172	1260	2393	2393*	1385
*Zea mays* ssp *mays*	373	180	114	169	117
*Oryza sativa* L.	340	271	92	95	86
*Solanum lycopersicum*	269	174	72	128	90
*Medicago truncatula*	149	99	40	45	40
*Glycine max*	61	39	30	30*	24
Total	6364	2023	2741	2866	1742

The number of EQ statements, genes, genotypes, and phenotypes they were associated with, for each species.

*#Genotypes equals # genes because no information on alleles was available for these species.

### Quantitative analysis of pairwise semantic phenotype similarity of genotypes across the entire dataset

To determine pairwise semantic phenotype similarity scores, we used the method described for mammalian genotypes [37] where phenotypes are represented by EQ statements that are then integrated using species-independent ontologies and a semantic similarity measure. That is, every phenotype (which is composed of one of more EQ statements) is compared to every other phenotype, and their similarity within the ontological graph is evaluated. For a pair of phenotypes to receive a score of 1, each phenotype would have to contain the same number of identical (or nearly identical) EQ statements. A score of 0 would mean that none of the EQ statements for either phenotype were similar. For the 8,213,956 possible pairs from the 2,866 genotypes, 548,888 (7%) of the genotype pairs yielded phenotype semantic similarity scores greater than zero. Score distributions, overall and on a per-species basis, are provided in the following sub-sections.

### Distribution of similarity scores

**Figure 2:**
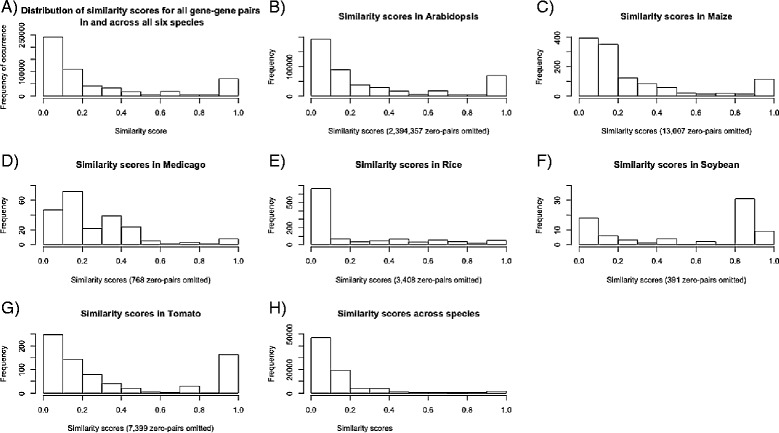
**Semantic similarity score distributions for inter- and intraspecific pairwise phenotype similarity.** When binning all semantic similarity scores across all species, 44% of semantic similarity scores indicate a relatively low phenotypic overlap between genes (semantic similarity range 0–0.1) while 13% show highly similar phenotypes (similarity score range 0.9-1) **(A)**. Distributions of intraspecific scores (pairwise scores where both genotypes belong to the same species) were similar to the overall distribution of scores **(B-H)**.

We calculated semantic similarity scores for 548,888 genotype pairs in the range of >0 – 1. A similarity score of 0 indicates no semantic overlap with respect to the phenotype, while a similarity score of 1 indicates an identical semantic phenotype description (and therefore equivalent sets of EQs). Figure 2A illustrates the distribution of semantic similarity scores for intra- as well as inter-species genotype pairs. For 13% (71,290) of the genotype pairs possessing a semantic similarity score, the score fell into the range 0.9 – 1 (not including the similarity of a genotype to itself, which is always 1). While 13% seems high, some of the nearly identical scores occur because of the limited availability of phenotype information for many genotypes. For example, if two genotypes are annotated with the same single EQ statement, the result is a semantic similarity score of one, even if in reality those mutant genotypes may have many more phenes that were not recorded. Only known phenes that were already curated from the scientific literature were assigned to genotypes, and our method cannot compensate for gaps in the literature (e.g., due to limitations in biological experiments). As the dataset grows, a better separation of genotypes with respect to their semantic phenotype similarity will be possible.

Almost half (241,042 = 44%) of the non-zero semantic similarity scores are below 0.1, indicating that many of the phenotypes show only a small overlap in their description. For example, the rice mutant DWARF4 (Os03g0227700 [45], allele osdwarf4-1) shows a similarity of 0.08 with the rice mutant MADS18 (Os07g0605200 [45]). This results from both genes being annotated with “dwarf”-related phenotypes but also possessing diverging annotations, such as *increased panicle number* for DWARF4 and *early flowering* for MADS18. It is not surprising that most of the phenotypes show at least some marginal overlap, as this is intrinsic to the aim of the study: making phenotypes comparable. This highlights the potential of the method, but, at the same time, raises the need for consistent, coherent, and complete phenotype annotations in order to computationally replicate the underlying biology and derive accurate predictions.

Although there are more complex scoring mechanisms that take frequency of EQ statements into consideration [23], we applied a Jaccard index that determines the overlap of phenes used in the phenotype descriptions. In an earlier study, it was shown that different types of semantic similarity measures do not differ much as long as the results are interpreted carefully [21]. In future work, we intend to investigate the applicability of alternative scoring methods, in combination with the development of benchmark sets for evaluation purposes.

### Species-specific distribution of scores

To obtain further insights into the distribution of similarity scores, we split similarity scores according to species. If both genotypes that were used to calculate the pairwise similarity score belong to the same species, we recorded the resulting similarity score only for this species. If both genotypes leading to a particular similarity score belong to different species, we recorded it as a cross-species score. The resulting seven similarity score distributions are illustrated in Figure 2B-H. Species-specific score distributions are mostly consistent with the overall score distribution (Figure 2A). There are some differences for soybean and Medicago, but this is likely due to the small sizes of the phenotype annotation sets in these species.

### Differences between the semantically-generated phenotype network and a manually derived phenotype grouping

A previous analysis of Arabidopsis used the same set of phenotypes and laid much of the groundwork for this present study [36]. Mutant phenotypes were categorized in a simplified, three-level hierarchy consisting of 4 groups divided into 11 classes and 42 subsets. Each gene was assigned to one of 11 phenotypic classes based on the developmental stage when the phenotype was first observed and what methods and conditions were used to detect it (see more details in Methods). Genes were also assigned to one or more of the 42 subsets, based on the nature of the phenotype (e.g., gametophyte defective, flowering time).

**Figure 3:**
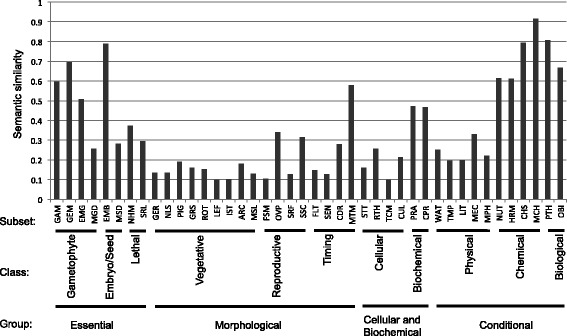
**Average semanitic similarity scores for previously derived groupings of Arabidopsis genotypes.** The average pairwise semantic similarity for subsets previously identified by [36] ranged from ~0.1 to ~0.9. Subsets are shown grouped by the classes and groups to which they belong.

To assess whether our results recapitulate those of [36], we calculated the average similarity scores for each of their classes (higher level grouping) and subsets (lower level grouping). Semantic similarity by class was greater than 0.3 for all classes except Vegetative, and ranged from 0.13 for Vegetative to 0.87 for Chemical and Biological (Additional file 3 and Figure 3). Average semantic similarity scores were lower and more variable across subsets, ranging from 0.10 for GEM (gametophyte, embryo defective) to 0.92 for OBI (other biological interactors), with 25 of 42 subsets having average scores less than 0.3 (Figure 3). Although there were indeed several classes and subsets that had good concordance with the semantic similarity scores, in general, semantic similarity scores within both classes and subsets were low (less than 0.5).

There could be several reasons for low semantic similarity scores within classes or subsets, such as annotations that are not ideally defined, a poor choice of semantic scoring mechanism, or classes/subsets that are too broadly defined and therefore contain a large variety of phenotypes. In general, we expected pairs of genes within the same subset to have lower semantic similarity scores than pairs of genes within the same class, because genes can belong to multiple subsets, but only a single class. If the phenotype of a gene has multiple phenes, that gene should to belong to multiple subsets, and unless two genes share all of the same phenes (and therefore belong to all of the same subsets), they would have a relatively low similarity score within each subset. Genes in the same class may have somewhat higher similarity scores, because classes aggregate several phenotypic subsets (Figure 3). However, they do not aggregate subsets from other classes (as semantic similarity does) and thus are unlikely to completely mirror semantic similarity scores.

Examination of two contrasting subsets, Flowering time (FLT) and Pathogens/Herbivores (PTH), can help to explain some of the agreement or disagreement between membership in a class or subset and degree of semantic similarity (Figure 3). The PTH subset seems to be more coherent with respect to phenotype annotations than the other groups, which suggests that PTH genes are not documented as having pleiotropic effects. In contrast, pairs of genes in the FLT subset have low average semantic similarity, suggesting that these genes are highly pleiotropic. Consistent with this, the PTH subset genes have on average 1.68 phenes whereas genes in the FLT subset have on average 3.99 phenes.

The categorical system devised by [36] has the distinct advantages of being more intuitive and not requiring an understanding of ontologies to make annotations or carry out an analysis of the data. However, the disadvantages are that category boundaries are sometimes somewhat arbitrary, very disparate phenotypes may be included in a single category (e.g., miscellaneous categories), and each phenotype may be forced into a single class. Although the class/subset classification can capture pleiotropic phenotypes, it does not provide a way to compare pleiotropic phenotypes of multiple genes the way semantic similarity scores based on collections of EQ statements does. In contrast, the ontology approach allows the grouping of phenotypes at any level of the ontology that may be appropriate for a particular analysis, while still allowing each observation (phene) to be separately annotated.

### Semantic similarity predicts participation in shared metabolic and regulatory pathways

It is a premise of this work that through computational analysis of EQ statements representing phenotypes, biological processes can be recapitulated, modeled, and even discovered. Were this to be true, one would expect, for example, that gene products in the same metabolic pathways would be annotated with EQ statements that are highly similar. To test this hypothesis, we used the PlantCyc project databases AraCyc (v 11.5) [46], Oryzacyc (v 1.0), SoyCyc (v 4.0) and CornCyc (v 4.0) as well as LycoCyc from SGN (v 3.3 *Solanum lycopersicum*) [47], and MedicCyc from the Noble Foundation [48]. One metabolic pathway that is well populated among those databases and for which our phenotype datasets have representation is the phenylpropanoid biosynthesis initial reactions of flavonoid biosynthesis. 3-hydroxy flavonoids, also called anthocyanins, are pigments. They serve to, e.g., attract pollinators and protect plants from UV-B damage [49,50].

For the gene products involved in the phenylpropanoid biosynthesis pathway – more specifically the initial reactions of flavonoid biosynthesis – we queried Plant PhenomeNET. The most informative query result came from maize, which had only the *c2* gene (*colorless2* converts 4-coumaryl-Coa to 2′, 4, 4′, 6′-tetrahydroxychalcone) curated into the phenylpropanoid biosynthesis initial reactions. When Plant PhenomeNet was queried with GRMZM2G422750 (the gene model identifier for *c2*) a number of maize genes associated with phenotypes were returned:

- *c2* GRMZM2G422750 similarity score 1 (identity: this is the query)
- *c1* GRMZM2G005066 similarity score 1
- *r1* GRMZM5G822829 similarity score 0.6666666667
- *b1* GRM similarity score 0.5

All three of the identified gene models are involved in the anthocyanin pathway of maize, which controls flavonoid synthesis (reviewed in [51]). More specifically, the gene products of the *c1, r1,* and *b1* loci activate genes in the anthocyanin pathway. This result: (1) indicates that reasoning across curated phenotypes in plants is capable of creating result sets that recapitulate well-characterized biological phenomena, (2) hints that for plant species that are not genetically well-characterized, the ontological reasoning approach to predicting phenotypic associations could assist in forward genetics approaches, and (3) highlights the potential use of reasoning across phenotypic ontological associations to prioritize high-quality data curation where data are missing from or complementary to repositories like the PlantCyc database.

Focusing on (2) – that the suggested approach can help with characterizing understudied species – the reasoning is as follows. Consider a poorly studied species with a number of mutant phenotypes that include an altered seed color phene. The phenotypes of this species would be described and codified using ontological representations. These phenotypic descriptions then could be used as queries to return genes from a well-characterized species (e.g., maize) with phenotypes that have high similarity to the phenotype in the poorly studied species. This result set could indicate to a researcher who is not an expert in pigment biology that the flavonoid and anthocyanin biosynthetic pathways and their regulators were of interest for determining which genes were responsible for the phenotype.

### Evaluation of phenotypic similarity across orthologs and gene families

#### Manual assessment of gene families

We were able to place 2,741 EQ-annotated genes (2,393 Arabidopsis, 30 soybean, 40 Medicago, 92 rice, 72 tomato, 114 maize) into 1,895 gene families, of which 460 families contain two or more genes annotated with EQ statements. The gene families, based on the Phytozome 10 Angiosperm-level families [52], generally contain both dicot and monocot representatives from the species in this study. Forty-two of the families contain between five and 12 genes with EQ statements, allowing us to assess how often homologous genes have similar functions. Further, there are 147 families that contain EQ statements from two or more species. These are of interest because it allows us to assess how often functions are conserved between orthologs.

For most families with multiple EQ-annotated genes, gene function is conserved or similar. For example, in the terpene synthase family (family 54585183, Additional files 4, 5 and 6), with 12 EQ-annotated genes from Arabidopsis, rice, and maize, all genes included aspects of “dwarf” phenotypes (quality “decreased height”, PATO:0000569). However, salient phenotypes in maize also include floral hermaphrodism, in contrast to the typical male and female floral separation in wild type domesticated maize. In the Flowering Locus T family (family 54614050, Additional files 4, 5 and 6), there are 12 EQ-annotated genes from five of our study species. All of the characterized mutant phenotypes involve floral development or photoperiod control.

We also observed gene families in which annotated phenotypes are quite different across orthologs. For example, in the family (54614050, Additional files 4, 5 and 6), a leucine-rich repeat, serine-threonine kinase family, the SUNN mutant in Medicago display extra root nodules, while the CLV1 mutant in Arabidopsis displays abnormal leaf phyllotaxy and altered floral morphology [53,54].

### Plant phenomeNET: a web interface for searching the plant dataset

We adapted PhenomeNET [37] to provide the results of the computational analysis of the plant data sets to the broader research community in an online form. Plant PhenomeNET is available via [39] and provides access to the genotypes of all six species that possess at least one EQ statement. For each genotype, a detailed genotype page provides information about similarity scores to any of the other genotypes as well as a link to an additional page providing the phenotype assigned by the curator and those inferred via the ontologies. We note here that similarity scores of 0 for genotype pairs are not reported in Plant PhenomeNET.

### Using plant phenomeNET -- Searching for *tasselseed1*

**Figure 4:**
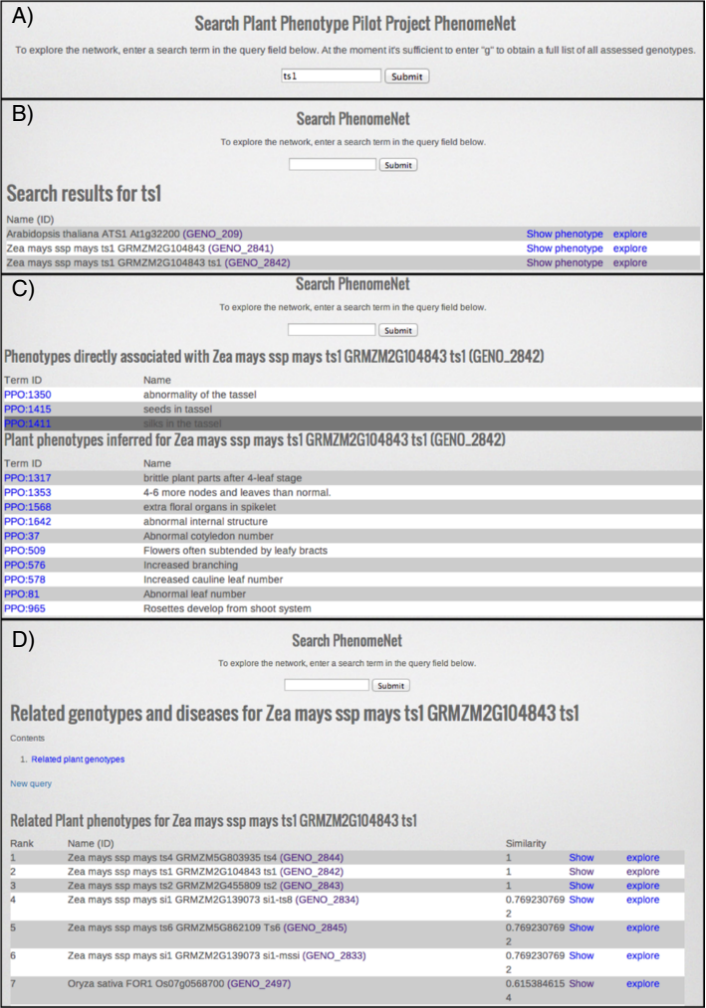
**This figures illustrates the usage of Plant PhenomeNET for the maize gene** ***mac1*** **.** After searching for the gene **(A)**, search results are returned **(B)** and assigned and inferred phenes are shown **(C)**, as well as semantically similar phenotypes from other genes **(D)**. See text for more details.

To illustrate the usage of Plant PhenomeNET, we provide an example search for *tasselseed1* (*ts1*) maize gene. The tassel of maize normally bears only male flowers, but in the *ts1* mutant, female flowers also develop in the spikelets born on the tassel. By entering “ts1” into the search box and submitting the form, we obtain a list of genes that all match the string “ts1” (for search query and results see: panel A and B of Figure 4). For the navigation from the search list, there are two options provided (see last two columns in panel B of Figure 4): one can either show the phenotype or explore phenotypically similar mutants.

By following the first link “show phenotypes”, the user obtains the assigned (top list, panel C, Figure 4) as well as the inferred EQ statements (bottom list, panel C, Figure 4) for the *ts1* gene. For example, the curator assigned an EQ statement for the “abnormality of the tassel” as part of the phenotype. One of the EQ statements that was inferred based on the ontology structure is “extra floral organs in spikelet”.

Following the second link to “explore” similar mutants, the user obtains an ordered list of phenotypically similar mutants with the most similar at the top and the least similar at the bottom (see panel D, Figure 4). Each of the mutants provided in the list can then be explored further with the links provided for navigation. One interesting case is presented in our list with the rice mutant FOR1, with the atomized statement “extra floral organs in the spikelet”. This example illustrates how important correctly assigned EQ statements are, and how, using inferred EQ statements, connections can made between mutants from different species.

## Conclusions

After defining a common method for semantic representation of plant phenotypes, we annotated 2,741 genotypes with 2,023 unique EQ statements. This represents the first cross-species plant data set that can readily be integrated with other data via shared ontologies. This use of ontologies to support phenotypic reasoning enables integration beyond plants and would enable generalized analyses to discover phenomena conserved across all domains of life as described in [55]. An example of such cross-domain inference is their finding that the pathways that underlie gravitropism sensing in Arabidopsis root tips are concordant with an inner ear defect in human developmental biology known as Waardberg syndrome [55].

In our initial computational analyses using semantic phenotype similarity scoring, we were able to identify gene sets that are functionally related, i.e. belonging to the same gene family or involved in the same pathway. The method described here can be readily applied to other plant species to suggest genes for analysis in under-studied species or crop wild relatives, or expanded to describe and compare phenotypes across diverse plant species for evolutionary analysis, as has been done for fish [17,56]. Because the ontologies used for comparisons are taxon-neutral, there are no restrictions to expanding this method to non-flowering plant models such as *Physcomitrella patens*, *Selaginella moellendorffi, or Ceratopteris* [57-59], and comparisons across widely divergent species (e.g., maize versus moss) may even reveal surprising conservation or co-option of gene function. Even for the species used in this initial study, there is much to learn about the relationships between genotype and phenotype, and we urge the continued curation and EQ-based annotation of plant phenotypes, to expand this data set and increase its utility. Because species- and clade-specific databases will need to curate and hold these statements, a standardized method for storing this data, preferably using a common database schema such as [60], should be implemented.

## Methods

### Generation of the lists of phenotypes for each plant species

Each of the model plant species represented in this analysis is supported by a database of genomic and other data. These databases are highly individualized, based on the differing needs of their community members. As such, the generation of a list of phenotypes associated with a mutant allele of a known gene was slightly different for each species.

#### Selection of Arabidopsis phenotypes

The Arabidopsis mutant phenotype dataset, first compiled by [36], includes ~2,400 genes with recessive mutant phenotypes for which the disrupted gene is known. Information for this dataset was previously gathered from: 1) a sequence-based map of genes with mutant phenotypes [61]; 2) the SeedGenes database of essential genes [62], as updated by [63]; 3) a list of genes associated with mutant phenotypes obtained from TAIR [64]; and 4) several thousand publications describing Arabidopsis mutant phenotypes retrieved from the Pubmed Database [65] using appropriate keywords (Arabidopsis, mutant(s), mutation(s), knockout, and null). Short, free-text phenotype descriptions found in column I of Supplemental Table S2 of [36] were used as the input for the Arabidopsis EQ statements. Genes with only a dominant, gain-of-function mutant phenotype [66] were generally excluded. Based on past work, the Arabidopsis phenotypes analyzed here are associated with sequenced genes but not with specific mutant alleles.

#### Selection of maize phenotypes

In the MaizeGDB database [31], maize phenotypes are associated with mutant alleles (variations) of genes based on a maize-specific controlled phenotype vocabulary, consisting of 1,088 phenotypes. Of the 1,088 phenotypes associated with mutant-defined loci, we removed continuous trait phenotypes (e.g., phenotypes that are attributable to quantitative trait loci or QTL), and several other types of phenotypes not likely to be relevant for this analysis, such as gel mobility of a protein on a starch gel. Of the remaining phenotypes, we selected only those associated with gene models (DNA sequences).

#### Selection of rice phenotypes

In order to create a list of rice mutants that were associated with known genes, data was combined from Gramene [34] and Oryzabase [32,67]. The Oryzabase file was quite large (about 4,800 traits/phenotypes listed, with about 1,600 of those associated with a known locus), while the Gramene list was smaller, with about 160 loci. The information from the two sets was combined and all the mutants with identifiers from both databases were cross-referenced to ensure there was no overlap or duplications. Many of the described mutants had to be eliminated from the master list as they were only described morphologically (i.e. not associated with a known locus or gene). For the remaining mutants, we combined all available phenotypic descriptions from the two sources.

#### Selection of soybean and Medicago phenotypes

Curated lists of phenotypes for these species are not available in public databases. Thus, in order to create lists of mutant genes in soybean and Medicago for this study, the primary literature was searched for phenotypes and their descriptions.

#### Selection of tomato phenotypes

Tomato loci with a known phenotype were selected from the Sol Genomics Network database (SGN) [15,35]. Phenotypes are associated with alleles, with some loci having multiple alleles with different phenotypes. The loci were curated manually based on previously described mutants [68] and literature curation of published tomato cloned genes with an associated phenotype. We included only loci with morphological or metabolic phenotypes, excluding isozyme alleles and loci that have a described phenotype but no associated gene sequence.

### Quality assurance across the entire data set

In order to provide consistency across species and allow for computational analysis of the entire phenotype data set, we developed a set of rules to define how the EQ statements should be constructed, and employed manual and automated quality checks to verify compliance with the rules. Manual checks determined if the EQ statements were made in a consistent manner across species. We did find consistency in most cases; however, minor inconsistencies have a relatively small effect, as the power of using hierarchical ontologies to describe phenotypes allows similar but not identical EQ statements to have high similarity scores.

Automated quality checks computationally verified the validity of the assigned EQ statements based on our pre-defined set of rules. An example of such a rule is a requirement that entities be represented with either PO (for structural) or GO IDs (for process phenotypes) and that the type of Quality chosen from PATO must match the Entity (i.e. a structure quality for a structure entity and a process quality for a process entity). Furthermore, the automated checks ensured that valid identifiers were used for each ontology term and that each term label matched its ID, which was useful for correcting typographical errors. More details on the rules we employed are provided in Additional file 7. The automated quality assurance was an iterative process in which the errors were removed continuously as the data set expanded. The data set comprising EQ statements from all six species (Additional file 1) successfully passed the automated checking procedure.

### Building a phenotype network using semantic similarities of gene pairs based on assigned EQ statements

The computational analysis relied on the representation of phenotypes as EQ statements. Each phenotype was represented as an affected entity that is further described with a quality. The application of EQ statements has been proven useful for cross-species gene function prediction, as well as pathway involvement and the identification of disease gene candidates [53,69]. As described by [19], species-specific phenes were decomposed into an affected Entity and Quality, and represented using species-independent ontologies. All the ontologies used here for the description of the phenotypes in any of the six species were downloaded on 15 March 2014 and converted to OWL EL. In addition to the ontologies, a set of logical definitions to connect plant structures with biological processes has been downloaded on 29 April 2013 and was also integrated with the ontologies (see Additional file 8). For further details on the applied ontologies see Table 1.

Once the ontologies were transferred into an OWL EL profile, they were combined into one ontology. We applied the method implemented in PhenomeNET [37], to represent the statements in OWL with:

$$ \mathrm{has}-\mathrm{part}\ \mathrm{some}\ \left(\mathrm{E}\ \mathrm{and}\ \mathrm{has}-\mathrm{quality}\ \mathrm{some}\ \mathrm{Q}\right) $$

where Entities and Qualities were used as defined by the curators. Following this approach generates one integrated ontology that then can be used to infer additional phenes using reasoning over the ontology. An inferred phene is an EQ statement that is an ancestor term of the assigned EQ statement. For example, the maize *mac1* (multiple archesporial cells1) gene was curated with an EQ statement named “Male and female infertility” and from the complete list of curator-assigned statements, and one additional EQ statement named “Complete sterility” was inferred.

To determine the semantic phenotype similarity of two genotypes (genotype A and B), a Jaccard index based on the binary vectors is calculated:

$$ \mathrm{simphen} = \left(\mathrm{P}\_\mathrm{geno}\_\mathrm{A}\ \cap\ \mathrm{P}\_\mathrm{geno}\_\mathrm{B}\right)\ /\ \left(\mathrm{P}\_\mathrm{geno}\_\mathrm{A} \cup \mathrm{P}\_\mathrm{geno}\_\mathrm{B}\right) $$

where P_geno_A represents the phenes of genotype A and P_geno_B represents the phenes of genotype B. Applying this scoring method, phenotype semantic similarity scores fall into the range [0, 1], with 0 indicating no overlap between phenotypes and 1 indicating identical phenotypes. Calculating the semantic similarity score for each possible combination of genotypes results in a 2,866 × 2,866 data matrix. Similarity scores > 0 are provided as Additional file 9. We note here that 10 EQ statements of Arabidopsis genotypes (<0.2% of total EQ statements) were excluded from the computational analysis, because they either needed further discussion among the curators due to the relations used to build the entity or include a term that was removed from the ontology in the period between curation and the computational analysis.

This matrix constituting a genotype network based on phenotype similarities was 1) compared to an existing, manually created phenotype-specific grouping of genes [36], and 2) used to assess gene function (see following sections and Results and Discussion). We note here that this scoring is highly dependent on the assigned EQ statements and that the annotations assigned to date are as complete as can be derived from existing findings. This means that for phenes that have not been tested yet, we assume that this phene is *absent*. With the growth of the data set, more detail will be added to the genotypes, which in consequence will improve the accuracy of semantic phenotype similarity scores and the representation of biological processes.

### Employed data and software

We downloaded all the ontologies from the OBO Foundry [70,71] or their respective download site (see Table 1), and used El Vira (version 0.2) [72] to transform ontologies from an OWL DL profile into an OWL EL profile. The application of OWL EL files facilitates faster reasoning over the combined ontologies and is consistent with the description of the method described for mammal data [37]. To integrate the individual annotation files along with the respective ontologies used in annotation into a single ontology, the Brain library version 1.5.2 was used to easily modify OWL EL ontologies [73]. All scripts required for the data analysis were implemented in Groovy (version 2.0.4) [74]. A copy of PhenomeNET was set up to hold the results of the computational analysis, which were uploaded using the PhenomeNET database scheme. Plant PhenomeNET is accessible from [39].

### Comparison of semantic similarity and an existing classification of plant phenotypes

For intraspecific comparison of Arabidopsis phenotypes, we used Table S2 from [36]. In this previous work, genes were sorted into a three-tiered hierarchy of phenotypes of groups, class, and subsets. Their classification system was designed for the specific purpose of defining the set of essential genes for an organism, and for this purpose it was not necessary to differentiate among phenotypes of different mutant alleles of the same gene. Genes were placed into a single group and class, prioritized by developmental stage when phenotypes are first observed and what methods and conditions are used to detect them. The lowest rank included phenotypes where detection required a biochemical assay or microscopic examination. When the phenotype of a weak allele was more informative or better characterized than the phenotype of a null allele, the assignment was made on the basis of the better-known phenotype (e.g., *fy* - null is *emb lethal* but known as *flowering time gene*). Genes were also assigned to one or more of 42 phenotypic subsets, such as shoot architecture, flowering time, miscellaneous seed defects, and temperature.

To carry out a comparison of the previous results to the present work, we rearranged the dataset from [36] so that each unique gene/subset combination was on a single row. Because genes could belong to multiple subsets, there were multiple rows per gene. We removed data for 82 genes that were in [36] but not included in the present study. We calculated average semantic similarity of the classes and subsets as the average of all pairs of genes where both genes were in the same class or subset.

### Pathway assessment based on phenotype network

The BioCyc databases for Arabidopsis (AraCyc version 11.5), maize (CornCyc version 4.0), rice (OryzaCyc version 1.0), and soybean (SoyCyc version 4.0) were downloaded from Plant Metabolic Network [45,75]. The database for tomato (LycoCyc version 3.3) was downloaded from the Sol Genomics Network [15,35], and the database for Medicago (MedicCyc version 2.0) was requested from and provided by The Samuel Roberts Noble Foundation [47,76].

To identify well-populated pathways across all six species, we divided the number of pathway steps catalyzed by a gene product for which a phenotype was included in our dataset by the average number of reactions in the pathway across the species examined (e.g., number of steps with a curated phenotype divided by number of total steps in the pathway). For instances where more than one gene encoded the enzyme responsible for a single step, that step was counted only once (i.e. the presence or absence of a gene encoding the enzyme was counted, not the number of genes encoding that step in a particular plant genome).

### Assessment of gene families using the phenotype network

Gene families are based on the Angiosperm-level families from the Phytozome10 release [50], accessed on August 13, 2014, as multiple-sequence alignments for each family. These gene family alignments included peptide sequences from 43 species, and comprised 29,803 gene families. From these alignments, we calculated HMM-based alignment models using hmmbuild (HMMer package version 3.1 r4562, Eddy, 2011 [77]). We then searched the peptide sequences from each of the seven species discussed in this paper, along with peptide sequences from *Amborella trichopoda* (to serve as an outgroup in phylogenies), against the gene family HMMs, using hmmscan (maximum E-value 1e-4), and then placed each sequence into the family of the top HMM match, giving a multi-fasta file for each gene family. The resulting family files were realigned to the respective HMM using hmmalign. Prior to generating phylogenetic trees, the resulting alignments were trimmed of non-aligning residues (as lower case characters in the output of hmmalign, indicating non-match-state residues in the HMM alignments). Phylogenetic trees were calculated using RAxML (raxmlHPC-PTHREADS-AVX, v. 8.0.26 [78]), using model PROTGAMMAAUTO. Analyses of EQ statements relative to gene families were conducted by generating combined EQ statements for each gene (concatenating multiple EQ statements into a single string separated by “;;”) and then joining these combined statements with genes. The resulting analyses are in Additional file 4. Alignments and phylogenetic trees are in Additional files 5 and 6, respectively.

Genome assembly and annotation versions used in these gene families were: *Glycine max* assembly and annotation version Wm82.a2.v1; *Medicago truncatula* assembly v 4.0v1; *Arabidopsis thaliana* v TAIR10; *Oryza sativa* Japonica (Nipponbare) assembly IRGSP-1.0, with the IRGSP-1.0 gene model names; *Zea mays* spp mays B75 RefGen v3, assembly annotation v 6a; *Lycopersicon esculentum* v iTAG2.3; *Amborella trichopoda* v 1.0.

## Endnotes

^a^Ontology term identifiers of the form PO:0000925 are shorthand for identifiers of the form http://purl.obolibrary.org/obo/PO_0009025. 

^b^Some relations in the Relation Ontology fall within the BFO namespace, because they are imported from the Basic Formal Ontology.

## Additional files

Additional file 1:
**All EQ statements curated for the six species.** All EQ statements in tabular form, with explanations of how to fill in each column. Additional file 2:
**Overlap among unique phenotypes for sets of species.** Unique phenotypes means that if one EQ statement is shared between two species, it is counted as overlap only once, no matter how often it occurs. Additional file 3:
**Average similarity scores for previously derived Arabidopsis genes grouped by class.** Classes follow [36]. Additional file 4:
**Gene families with EQ statements.** An Excel file with gene family membership by species, concatenated EQ statements for genes with EQ annotations, and gene family descriptions. Additional file 5:
**Gene family alignments.** An archived, compressed directory of the multi-fasta alignments (text files) for the 1,985 gene families with EQ statements from this study. Access using “tar -xzf alignments_w_EQs.tar.gz” and then with an alignment viewer or standard text editor. Additional file 6:
**Gene families with EQ statements.** An archived, compressed directory of the phylogenetic reconstructions (“trees”), calculated from the alignments in Additional file 5. Tree files are in Phylip/Newick format. Access using “tar -xzf trees_w_EQs.tar.gz” and then with a phylogenetic tree viewer. Additional file 7:
**List of error checks for the EQ statements.**
Additional file 8:
**Logical definitions for biological processes in plants.** A subset of logical definitions built to connect biological processes with plant structures were used as part of the computational analysis. While the logical definitions are now part of GO, we used an earlier independent version provided here as an OWL file, which can be opened in a text editor or OWL editor. Additional file 9:
**Similarity scores of genotype pairs with similarity >0.**

## Abbreviations

EQ: Entity-Quality
GO: Gene Ontology
MaizeGDB: Maize Genetics and Genomics Database
OWL: Web Ontology Language
PATO: Phenotype and Trait Ontology
PO: Plant Ontology
QTL: Quantitative trait locus (or Loci)
RO: Relation Ontology
SGN: Sol Genomics Network
TAIR: The arabidopsis information resource
