# Supplementary figures and images for: An ontology approach to comparative phenomics in plants
Source: Plant Methods. 2015 Feb 25;11:10. doi: 10.1186/s13007-015-0053-y (PMC4359497; doi:10.1186/s13007-015-0053-y)

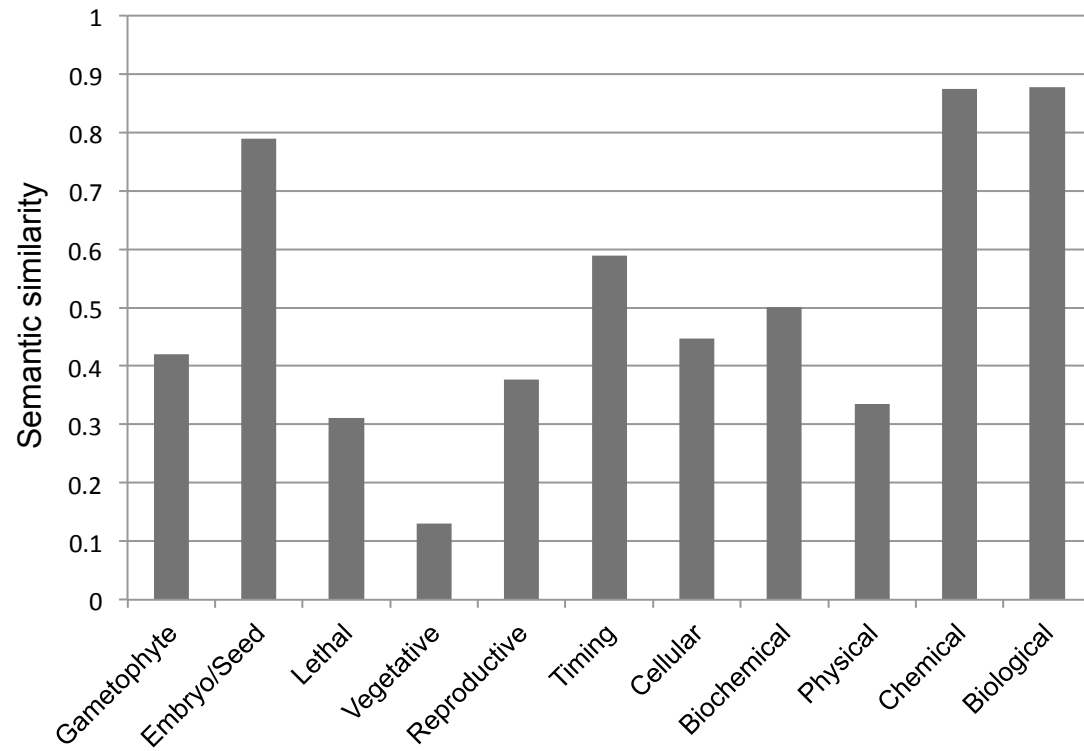

Supplement: Additional file 3: — Average similarity scores for previously derived Arabidopsis genes grouped by class. Classes follow [36]. [file 13007_2015_53_MOESM3_ESM.pdf]
