# Supplementary material for: An ontology approach to comparative phenomics in plants
Source: Plant Methods. 2015 Feb 25;11:10. doi: 10.1186/s13007-015-0053-y (PMC4359497; doi:10.1186/s13007-015-0053-y)
Supplement: Additional file 7: — List of error checks for the EQ statements. [file 13007_2015_53_MOESM7_ESM.pdf]

Additional File 7: List of error checks used for plant phenotype EQ statements

Rule	in place	not in place	code	comment
At least primary entity1 and quality must be set	x		PE1QM	reported if either primary entity1 wrong id or quality null
Primary entity1 (ID) is a valid GO or PO ID	x		PE1IV	reported when first primary entity invalid
Primary entity2 (ID) is a valid GO, PO or ChEBI ID (or null)	x		PE2IV	reported when second primary entity invalid
Secondary entity1 (ID) is a valid GO, PO, ChEBI or NCBI Taxonomy ID (or null)	x		SE1IV	reported when first secondary entity invalid
Secondary entity2 (ID) is a valid GO, PO or ChEBI ID (or null)	x		SE2IV	reported when second secondary entity invalid
Quality (ID) is a valid PATO ID	x		QIV	reported when quality ID not valid PATO ID
Quality (ID) is not PATO:0000460 (abnormal)	x		QA	reported when quality is only "abnormal"
If primary entity1 (ID) is a GO biological process or molecular function ID, quality (ID) is a child of 'PATO:process quality'	x		QNP	will be reported if quality is not part of PATO:process quality
If quality (ID) is in the PATO relational slim, secondary entity 1 (ID) must not be null. Also, if secondary entity (ID) is not null, quality (ID) is in the relational slim.	x		SRE1	
If secondary entity 1 (ID) not null, PATO quality must be in relational_slim		x	E1SR	will be reported if secondary entity 1 set but quality not in relational_slim
Quality has to be in branch of "physical object quality" or "process quality" in ontology	x		QYIV	will be reported if quality not in either branch
Qualifier: everything under "qualitative" is allowed but nothing from branch "decreased/increased quality" (these concepts encompass	x		QFIV	reported when qualifier isn't in the allowed subset of PATO concepts

quality and qualifier in one concept)				
If primary entity2 (ID) is null, relation_to (previous column) is null.	x		PERM	reported when related but no e2 for primary entity
If both primary entity1 (ID) and primary entity2 (ID) are PO IDs or GO cellular component IDs, relation_to = part_of or adjacent_to.	x		PEIR	reported when relation used is different from part_of or adjacent_to
If primary entity1 (ID) is a GO biological process or molecular function ID and primary entity2 (ID) is a PO ID or GO cellular component ID, relation_to = occurs_in.	x		PEIR2	reported when relation differs from "occurs_in"
If primary entity2 (ID) is a ChEBI ID, has_participant is allowed.	x		PECR	reported when prim e2 is ChEBI but relationship is wrong
If secondary entity2 (ID) is not null, relation_to (previous column) is not null	x		SERM	will be reported if relation is not set for secondary entity but two parts of it are reported
Developmental stage (ID) is equal to PO:0009012: plant structure development stage or a child of this term (or null).	x		DEVIV	will be reported if PO is not under structure dev stage or term as such
Condition (ID) is a valid EO ID (or null)	x		CIV	will be reported if invalid EO
No question marks in any field	x		QO	will be reported when ? occurs in any cell of a row
The ontology term ID and name match for each ontology concept id and term in following columns of the file	x		TMM	will be reported if mismatch of id and name occurs (maybe reported multiple times for same EQ if multiple labels should be wrong)
